# Comparison of Artificial Neural Network (ANN) Model Development Methods for Prediction of Macroinvertebrate Communities in the Zwalm River Basin in Flanders, Belgium

**DOI:** 10.1100/tsw.2002.79

**Published:** 2002-01-12

**Authors:** Andy P. Dedecker, Peter L.M. Goethals, Niels De Pauw

**Affiliations:** Laboratory of Environmental Toxicology and Aquatic Ecology, Ghent University, J. Plateaustraat 22, B-9000 Gent, Belgium

**Keywords:** neural networks, model validation, ecological modeling, sensitivity analyses

## Abstract

Modelling has become an interesting tool to support decision making in water management. River ecosystem modelling methods have improved substantially during recent years. New concepts, such as artificial neural networks, fuzzy logic, evolutionary algorithms, chaos and fractals, cellular automata, etc., are being more commonly used to analyse ecosystem databases and to make predictions for river management purposes. In this context, artificial neural networks were applied to predict macroinvertebrate communities in the Zwalm River basin (Flanders, Belgium). Structural characteristics (meandering, substrate type, flow velocity) and physical and chemical variables (dissolved oxygen, pH) were used as predictive variables to predict the presence or absence of macroinvertebrate taxa in the headwaters and brooks of the Zwalm River basin. Special interest was paid to the frequency of occurrence of the taxa as well as the selection of the predictors and variables to be predicted on the prediction reliability of the developed models. Sensitivity analyses allowed us to study the impact of the predictive variables on the prediction of presence or absence of macroinvertebrate taxa and to define which variables are the most influential in determining the neural network outputs.

### INTRODUCTION

Nowadays, numerous models are being used in aquatic ecology. Many of these models describe the macroinvertebrate communities. These organisms are the most used indicator group for biological water quality assessment[1]. Examples of models based on macroinvertebrate communities are the River Invertebrate Prediction and Classification System (RIVPACS)[2], the

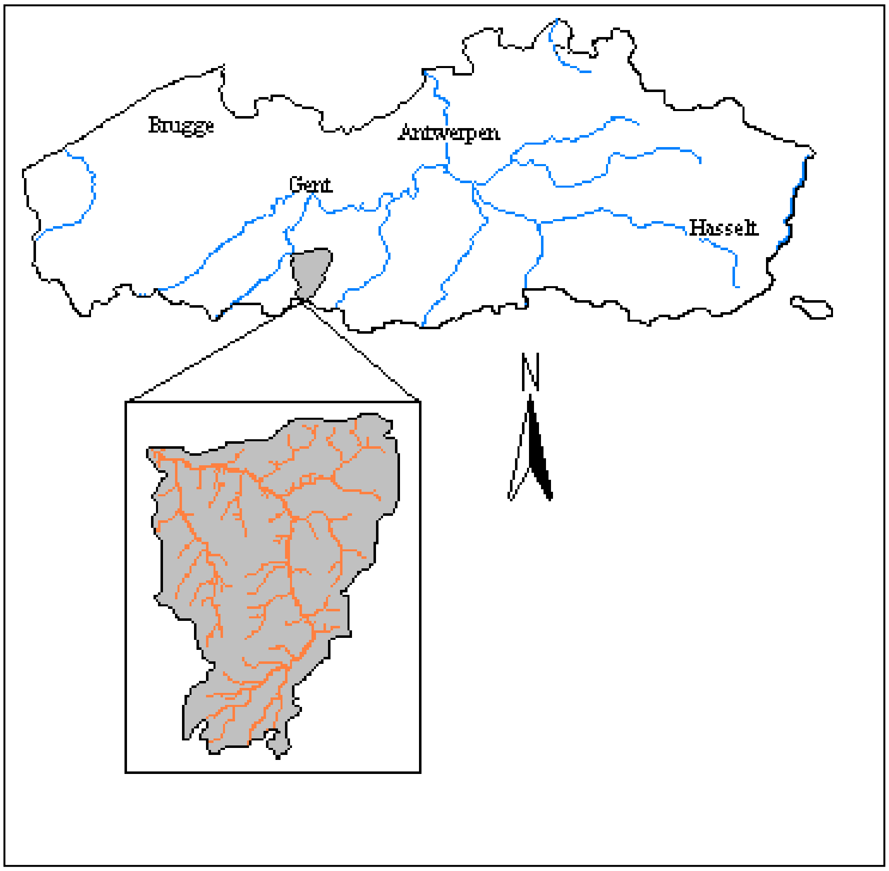

**FIGURE 1**. The Zwalm River basin in Flanders, Belgium.

Australian River Assessment System (AusRivAS)[3], and the Benthic Assessment of Sediment (BEAST)[4]. These models, based on multivariate statistics, are criticized because of their complexity[5]. During recent years new techniques such as artificial neural networks (ANN)[6], fuzzy logic[7], and evolutionary algorithms[8] are being more commonly used to analyse ecosystem databases and to make predictions for river management purposes[9]. In this context, ANN were applied to predict macroinvertebrate communities in the Zwalm River basin (Flanders, Belgium)[10].

### EXPERIMENTAL METHODS AND PROCEDURES

The Zwalm River basin was selected as the study area. The Zwalm River basin (117 $km^2$) is, according to the Flemish Hydrographic Atlas, part of the hydrographic basin of the Scheldt River (Fig. 1)[11]. The Zwalm River itself has a length of 22 km. The average water flow at Nederzwalm, very near the upper Scheldt is about 1 $m^3 s^{-1}$. It has an irregular regime, with low values in the summer (minima lower than 0.3 $m^3 s^{-1}$) and relatively high values in rainy periods (maxima up to 4.7 $m^3 s^{-1}$)[12]. The water quality in the Zwalm River basin substantially improved during the year 1999 due to investments in sewage and wastewater treatment plants over the last year[13]. Nonetheless, many parts of the river are still polluted by untreated urban wastewater input and by diffuse pollution originating from agricultural activities.

In total, 60 measuring sites were monitored in the Zwalm River basin (Fig. 2). The data were gathered on structural (meandering, substrate type, flow velocity, etc.) and physical and

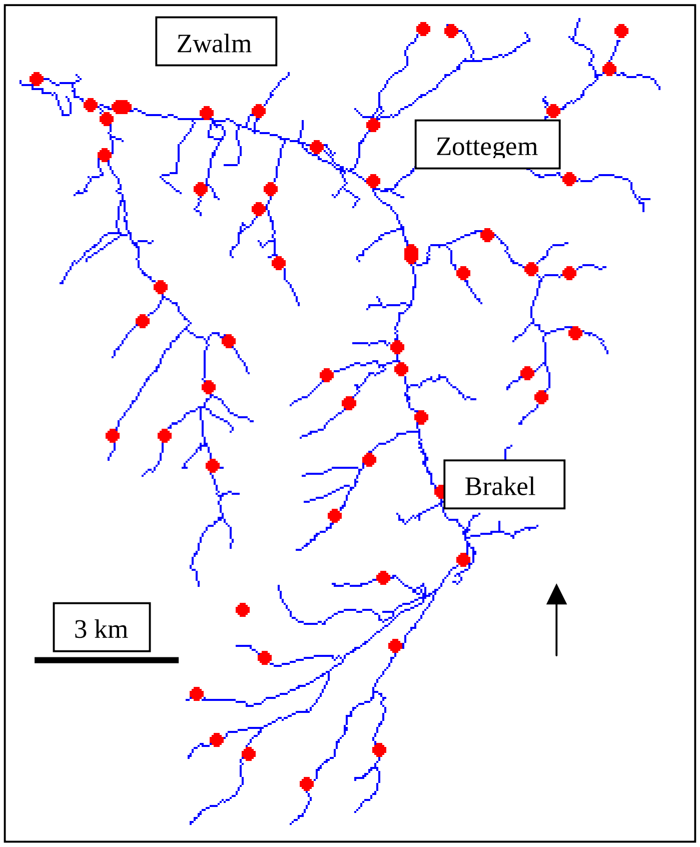

**FIGURE 2**. Selected sampling sites in the Zwalm River basin.

**TABLE 1**
**Input Variables and Units used in the ANN Model**

| **Variables** | *Units* |
|---|---|
| Temperature | °C |
| pH | - log [$H^+$] |
| Conductivity | µS/cm |
| Suspended solids | mg/l |
| Dissolved oxygen | mg/l |
| Water level | cm |
| Fraction of pebbles | % |
| Shade | % |
| Water plants | present or absent |
| Width | cm |
| Flow velocity | m/s |
| Meandering | 6 classes[14] |
| Hollow river beds | 6 classes[14] |
| Deep/shallow variation | 6 classes[14] |
| Artificial embankment structures | 3 classes[14] |

chemical characteristics (dissolved oxygen, pH, etc.) and the macroinvertebrate composition were collected (Table 1). The structural characteristics and physical-chemical variables were used as inputs for the ANN models to predict the presence or absence of macroinvertebrate taxa in the headwaters and brooks of the Zwalm River basin. Structural characteristics were visually

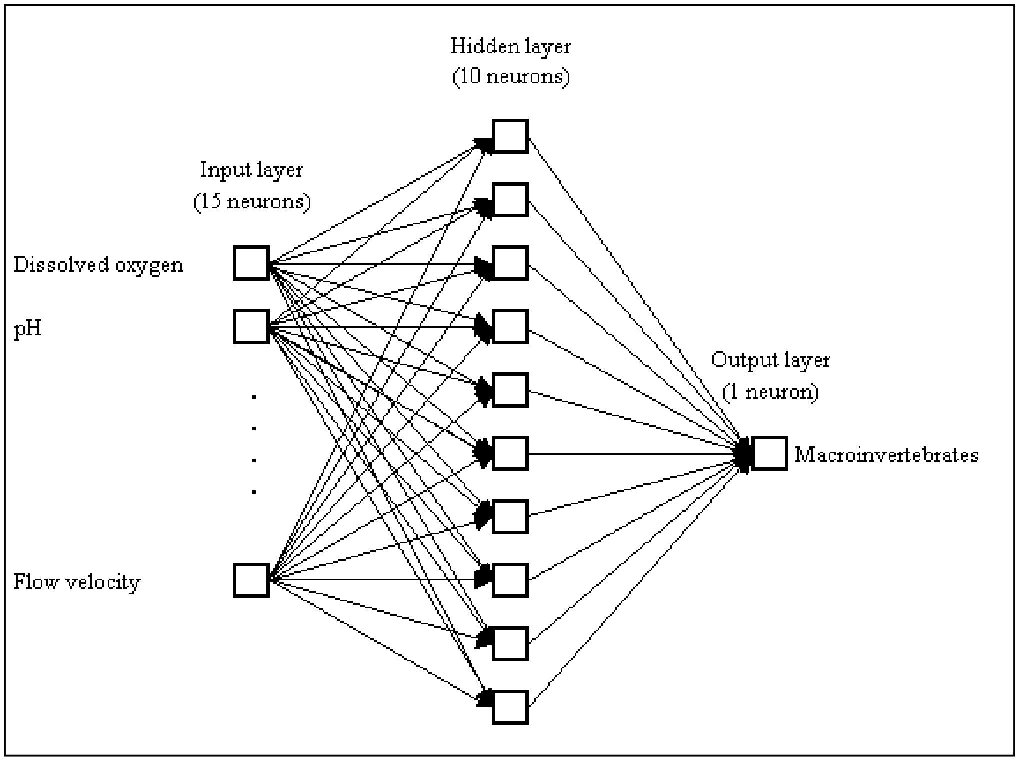

**FIGURE 3**. Scheme of the ANN.

monitored[14]. Flow velocity was determined by timing the transport of a float over a distance of 10 m. Field measurements were made for temperature and dissolved oxygen (OXI 330/SET), pH (Jenway 071), and conductivity (WTW LF 90). Suspended solids were measured spectrophotometrically in the laboratory[14].

Macroinvertebrates were collected by means of a standard handnet[15] during 5-min kick sampling within a river stretch of 10 m. The objective of the sampling was to collect the most representative diversity of the macroinvertebrates on the examined site[16]. For use in the different models, the absence or presence of macroinvertebrate taxa was respectively represented by 0 or 1.

ANN models are a modelling technique from the field of artificial intelligence. In this paper, backpropagation ANNs[17] were used. With this type of ANN, a set of training examples, consisting of an input and an output vector, was presented to the network. The backpropagation network determines its own parameters with specific training algorithms. After training, the neural network is able to calculate an output vector for any new input vector. The neural network was implemented with the neural network extension of the software package MATLAB 5.3 for MS Windows™[18]. The model validation was based on splitting the data set in a training and validation set of respectively 40 and 20 patterns. Also, threefold and fourfold cross-validation was applied, as described by Witten and Frank[19]. Several optimisation studies were carried out to select the best model configuration[14]. The best neural network consisted of one hidden layer and ten neurons, with tangential and logarithmic sigmoid transfer functions and gradient descending with momentum and adaptive learning rate backpropagation as training algorithm[18]. A scheme of the applied neural network is shown in Fig. 3.

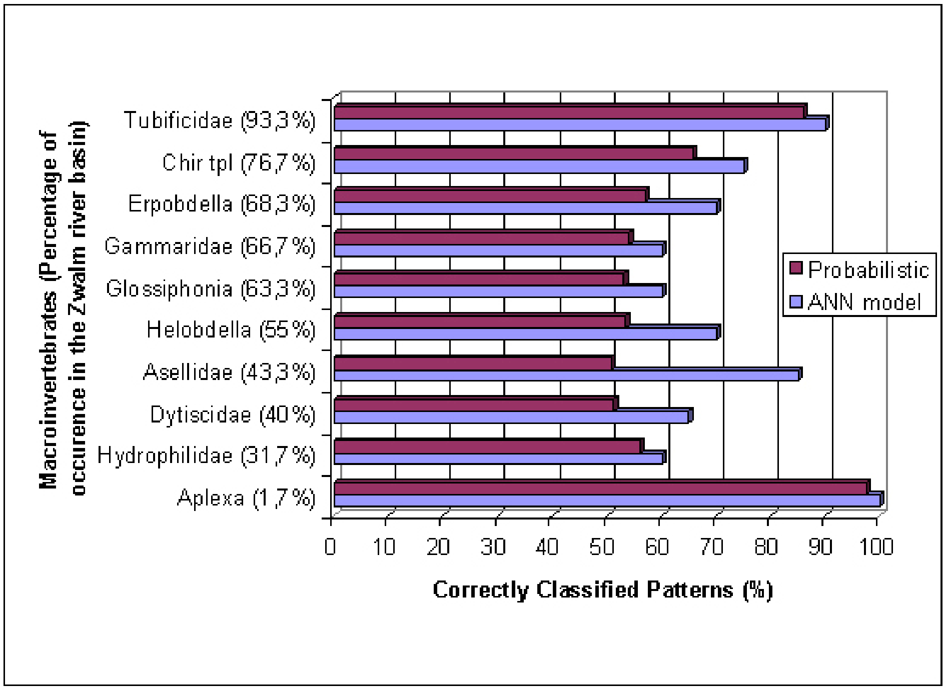

**FIGURE 4.** Prediction of ten macroinvertebrate taxa based on ANN and compared with an ordinary probabilistic guess (Chir tpl = Chironomidae *thummi-plumosus*).

## RESULTS AND DISCUSSION

### Training with a Dataset Consisting of 40 Patterns and Validation Set with 20 Patterns

After selecting the best model configuration, the presence (= 1) or absence (= 0) for ten macroinvertebrate taxa was predicted. The model has been evaluated using the percentage of Correctly Classified Patterns (CCP). In Fig. 4 one can clearly see that ANN make better predictions compared to ordinary probabilistic guesses. These probabilistic guesses are calculated as follows:

(the frequency of presence in the validation set) × (the probability of predicting ad random the presence based on the training set)
+
(the frequency of absence in the validation set) × (the probability of predicting ad random the absence based on the training set)

Several authors also proved that ANN are good alternatives for Multiple Regression (MR)[20]. Another typical feature of ANN models is that the reliability of the model is the highest for very common (e.g., Tubificidae) and extremely rare taxa (e.g., *Aplexa*). The real problem is that there is not enough information to allow the ANN to learn when frequent species are absent and when rare species are present. In this way ANN models tend to “learn” that very common species are always present and very rare are always absent.

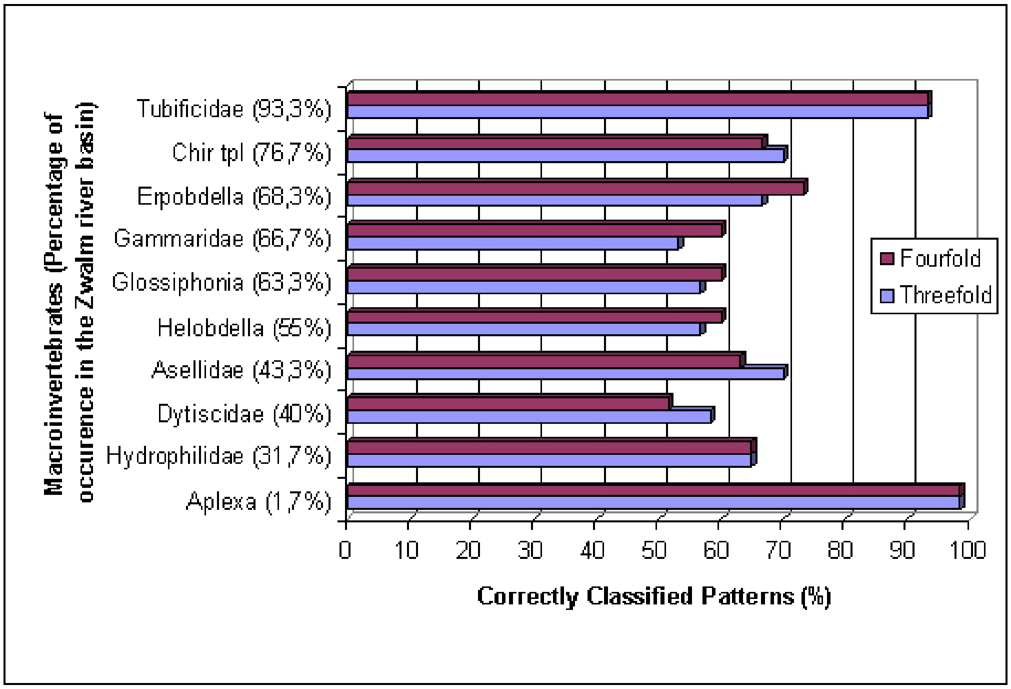

**FIGURE 5.** Prediction of ten macroinvertebrate taxa based on threefold and fourfold cross-validation (Chir tpl = Chironomidae *thummi-plumosus*).

### ANN Model Development with Threefold and Fourfold Cross-Validation

Cross-validation is appropriate when the amount of data for model development is quite small[19]. In cross-validation, one decides on a fixed number of folds, or partitions of the data. Suppose one is using three partitions, as in Fig. 5. Then the data are split into three equal partitions, and each in turn is used for validation while the remainder is used for training. That is, use two thirds for training and one third for validation, and repeat the procedure three times so that in the end every instance has been used exactly once for validation. The same procedure counts for fourfold cross-validation. From Fig. 5 one can see for cross-validation the same feature as from Fig. 4 where cross-validation has not been used. Again, the reliability of the model was highest for very common (e.g., Tubificidae) and extremely rare taxa (e.g., *Aplexa*). Also, there was no difference between the CCP of threefold and fourfold cross-validation. Both methods gave similar results on the used dataset.

### Comparison 40/20 Training/Validation with Cross-Validation for ANN Model Development

Comparison between threefold cross-validation and validation with 20 patterns without cross-validation is shown in Fig. 6. Because there were only 60 sites monitored, which is rather small, one should expect cross-validation to have a better performance[19]. However, from Fig. 6 one cannot decide whether this procedure of validation is better than the other or not.

### Sensitivity Analyses

A disadvantage of ANN could be their lack of explanations regarding the relative importance of each independent variable considered. In ecology, however, it is useful to know the magnitude of

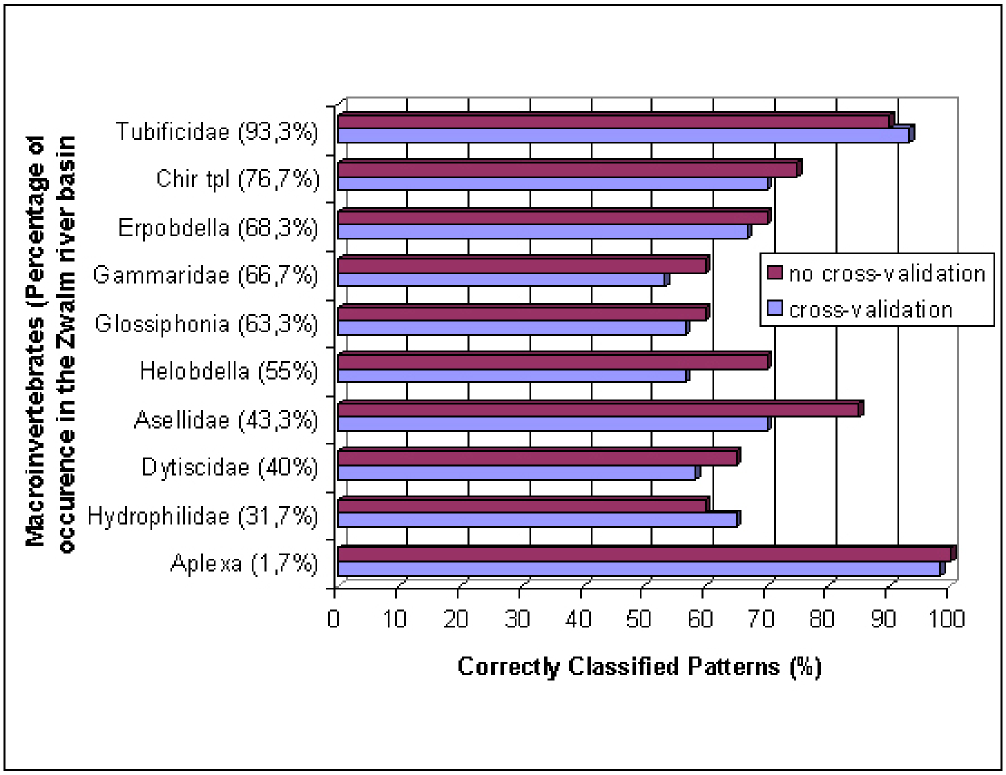

**FIGURE 6**. Prediction of macroinvertebrate taxa based on threefold cross-validation and validation with 20 patterns (Chir tpl = Chironomidae *thummi-plumosus*).

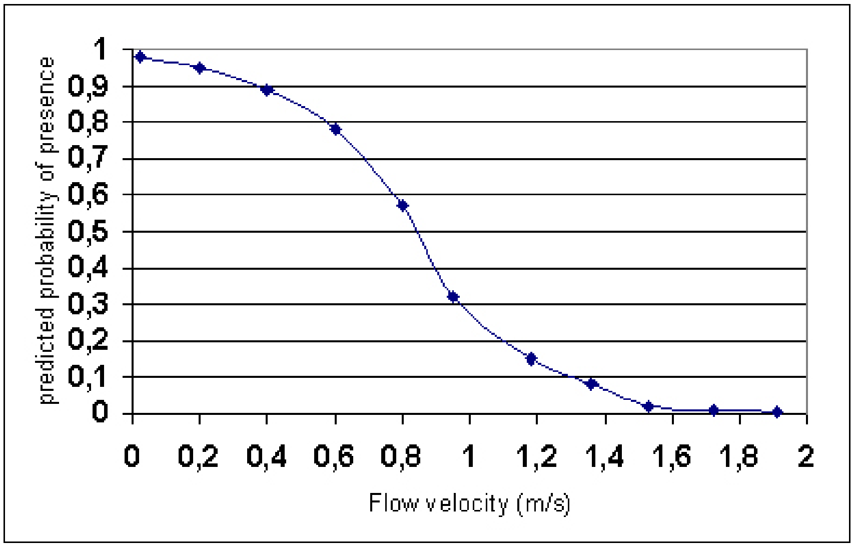

**FIGURE 7.** The impact of flow velocity on the probability of presence of Hydrophilidae.

impacts of each variable. In this work, an experimental approach has been used to determine the response of the model to each of the input variables separately by applying a range of variation of a single independent variable to the model, while the others are held constant (at the average value of the database). In this way, one is able to determine the impact of the variable on the presence or absence of a specific taxon. From Fig. 7 and Fig. 8 one can conclude for example that

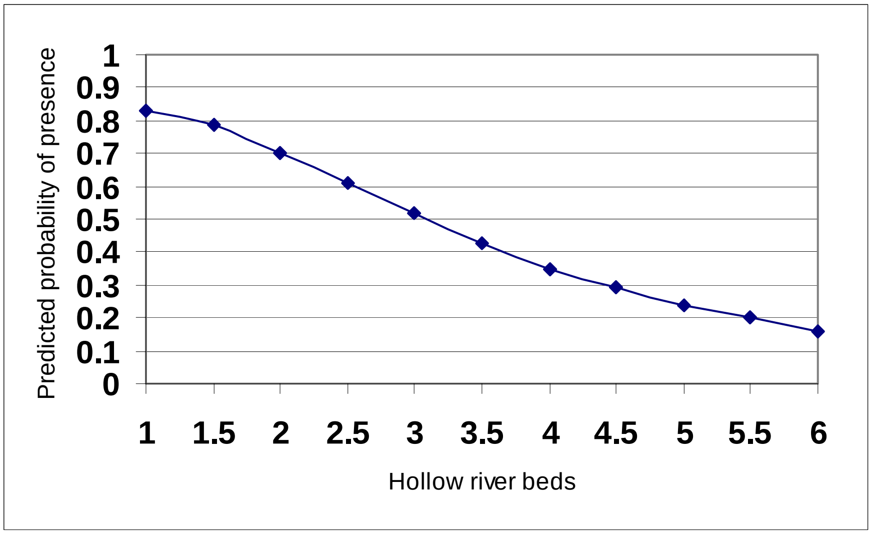

**FIGURE 8.** The impact of hollow river beds on the probability of presence of Hydrophilidae.

Hydrophilidae prefer slow-flowing waters where hollow river beds are not frequent. So sensitivity analyses provide some insight into the habitat preference of the taxa, which delivers relevant information for river ecosystem management. Sensitivity analyses can also be used in ecotoxicology or to meet environmental standards.

### CONCLUSION

This paper showed that artificial neural networks can provide useful predictions about the occurrence of some macroinvertebrate taxa in the Zwalm River basin. A feature of the ANN models was that the reliability of the ANN models was highest for very common and extremely rare taxa. Although a small dataset was used, cross-validation did not result in a better reliability. Last but not least, the sensitivity analyses provided some insight in the habitat preference of all taxa, which delivers relevant information for river ecosystem management.
